# Supplementary material for: A monolithic immersion metalens for imaging solid-state quantum emitters
Source: Nat Commun. 2019 Jun 3;10:2392. doi: 10.1038/s41467-019-10238-5 (PMC6546684; doi:10.1038/s41467-019-10238-5)
Supplement: Supplementary file 2 — Supplementary Information [file 41467_2019_10238_MOESM2_ESM.pdf]

Supplementary Information for:

A Monolithic Immersion Metalens for Imaging Solid-State Quantum Emitters

Huang *et al.*

Supplementary Note 1. COMPARISON OF A METALENS TO A SOLID IMMERSION LENS

To illustrate the advantages of a metalens design for coupling to quantum emitters, we compare its operation to that of a solid immersion lens (SIL) in Fig. 1. While a SIL corrects for the losses caused by total-internal-reflection and spherical aberrations when imaging through a planar surface, a second optic with a large NA (typically a microscope objective) is required to collimate the diverging dipole emission pattern produced by a quantum emitter (Fig. 1**a**). In contrast, our metalens produces a collimated output (Fig. 1**b**) that can be directly coupled into a fiber or other low-NA collection optics, as shown in Fig. 3**b** of the main text. Thus a metalens, through careful design of its phase profile, is capable of overcoming total-internal-reflection and spherical aberration losses in a similar manner to a SIL while combining the functionality of both a SIL and a microscope objective by acting as an immersion optic.

To compare our metalens design with conventional imaging geometries, we examine the NA and its corresponding acceptance angle in diamond, θ_{d}, in Table 1.

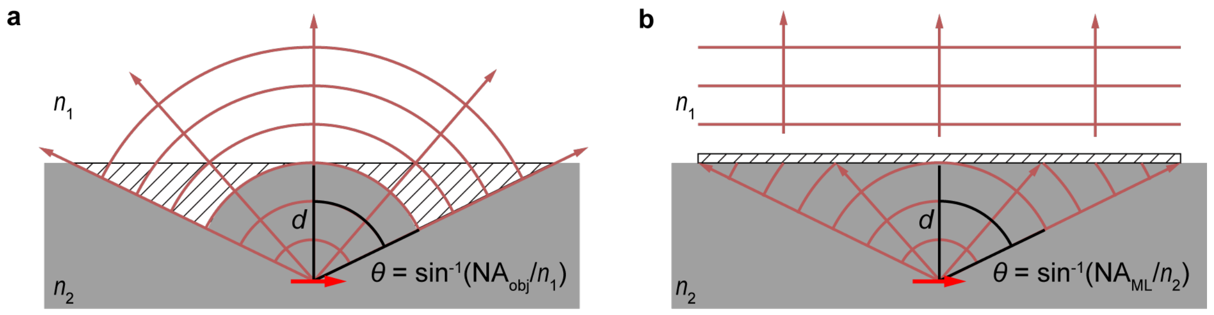

Supplementary Figure 1. **Comparison between Metalens and a Solid Immersion Lens.** (**a**) Metalens (**b**) Solid Immersion Lens.

	Air obj.	Oil obj.	SIL w/ air obj.	Immersion metalens
$\text{NA} = n_\text{d} \sin\theta_\text{d}$	0.95	1.40	2.16	1.10
$\sin\theta_\text{d} = \frac{\text{NA}}{n_\text{d}}$	0.40	0.58	0.90	0.46
θ_d	23.2°	35.7°	64.2°	27.3°

Supplementary Table 1. Comparison of NA and collection angle, θ_d for different imaging optics. The host material is diamond with $n_\text{D} = 2.4$. The NA of the metalens is measured to be $1.10^{+0.12}_{-0.09}$ at $\lambda = 700\,\text{nm}$ (Fig.3**c** in the main text)

Table 1 demonstrates that in terms of geometric efficiency, measured either by the acceptance angle or by the NA of the collection geometry, the demonstrated metalens outperforms the best-case air objective when imaging through a planar surface. While our initial demonstration does not yet reach the maximum collection achievable using a SIL-objective combination, metalenses with NA = 0.99 in air[1] have been demonstrated, suggesting that a comparable immersion metalens with $\text{NA} = 0.99 \cdot n_d = 2.38$ should be achievable.

Ultimately, the most important performance metric for both SILs and metalenses is the photon collection efficiency. Detailed measurements and calculations for the metalens coupling efficiency are included in Supplementary Note 6. Despite much room for improvement in the metalens coupling efficiency and NA, the demonstrated photon collection efficiency of the metalens-fiber combination already rivals the performance of a typical confocal microscope using a high-NA objective. Considering the additional mechanical stability and reduced number of optical components in a fiber-coupled system, it should eventually be possible to match the performance of the best free-space optical designs using SILs.

In addition to its optical performance, the processes used to fabricate the metalens are more scalable than those used for a SIL, as SILs require 3D patterning typically achieved by focused ion beam[2,3], laser micromachinging[4], or some form of grayscale lithography[5], with tight alignment tolerance to the NV due to the limited field-of-view of the SIL. The SIL field-of-view diameter[3] is limited by the refractive index of host material:

$$d_\text{FOV} < \sqrt{\frac{2r\lambda}{n_2(n_2-1)}} \tag{S1}$$

whereas a metalens has a field-of-view diameter of $2r$, with r being the radius of the SIL and the metalens. The simple Fresnel phase profile used in our demonstration exhibits coma

aberration[6] and a restricted field-of-view of about $2\,\mu m$[7], which may degrade the collection efficiency for an emitter that is not centered at the optical axis, and has a relatively short focal length of $\sim 20\,\mu m$; however, the alignment tolerance is much more relaxed than the requirement of $< 1\,\mu m$ alignment accuracy needed for the SILs reported in Jamali *et al.*[3], which are subjected to further aberrations from fabrication imperfections and would in turn require additional corrective optics.

The collection angle of a SIL is determined by the microscope objective used to collimate the output light, while the collection angle of a metalens is limited by the design of the phase profile. Assuming that a dielectric metalens can be designed with the same collection angle as a microscope objective, the surface area that requires patterning, $A_{\mathrm{ML}} = \pi d^2 \tan^2\theta$, (hatched lines, Fig. 1**a**) to fabricate a metalens that collects light from a quantum emitter located at a depth, d, below the substrate surface with a collection angle, θ, is identical to the area occupied by a SIL at the substrate surface. However, fabrication of a SIL requires the removal of a volume of material, $V_{\mathrm{SIL}} = \frac{\pi d^3}{3}\left(\tan^2\theta + 2\cos\theta - 2\right)$ (hatched lines, Fig. 1**b**). Since the metalens is defined by lithographic patterning, the time required to write the metalens pattern scales with the pattern area, which scales with d^2, whereas the time required to fabricate a SIL scales with d^3. More novel approaches to fabricating metasurlenses, such as nanoimprint lithography, could also be implemented to eliminate size-dependence in fabrication time.

Beyond the implications for packaging fiber-coupled quantum emitters, there are opportunities to incorporate other optical elements with the metalens, since we have the freedom to design arbitrary phase profiles.

Supplementary Note 2. SUBWAVELENGTH ELEMENT SIMULATIONS

Comparisons of our rigorous coupled-wave analysis (RCWA) MATLAB code to open source planewave expansion[8] and RCWA software[9] are shown in Fig. 2 to verify the accuracy of our calculations. As described in the methods section of the main text, the Bloch-mode effective index calculated by solving for the eigenvalues of Maxwell's equations in a truncated planewave basis with implicit periodic boundary conditions is shown in Fig. 2**a**. The effective index of the lowest-order HE_{11} mode supported by an isolated pillar is also shown for comparison. The corresponding normal-incidence phase shift for $1.0\,\mu m$-high

pillars on a homogeneous diamond substrate, calculated by $\phi(d) = \angle t(d)$, is shown in Fig. 2**b**. Among the advantages of our metasurface design is its high transmission efficiency. Since

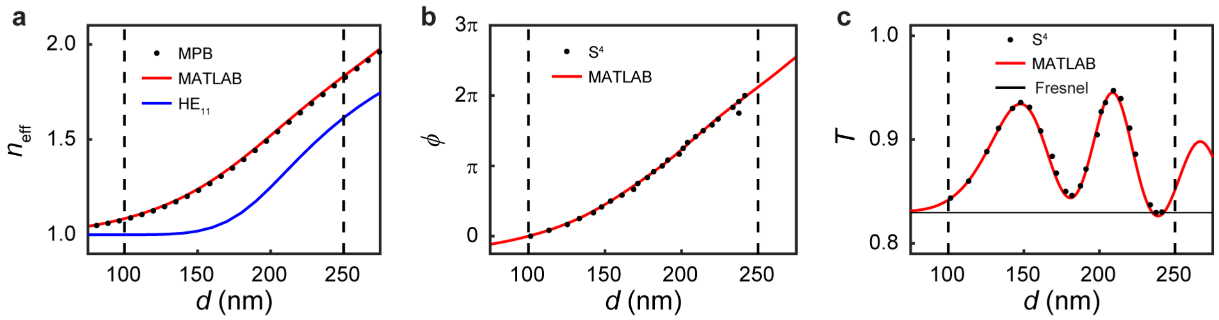

Supplementary Figure 2. **Metasurface element design.** Simulations of (**a**) effective refractive index, n_{eff}, (**b**) optical transmission phase shift, ϕ, and (**c**) transmission efficiency as a function of pillar diameter, d, for normal incidence at $\lambda = 700\,\mathrm{nm}$ on a $\Lambda = 300\,\mathrm{nm}$ grid. The effective index of an isolated waveguide is also shown in (**a**) and the theoretical transmission efficiency of a planar air/diamond at normal incidence is shown in (**c**). Black-dashed lines indicate the range of d used for our metalens design. Comparisons of our MATLAB code, based on ref.[10], to open source simulation tools S^4 (see ref.[9]) and MIT Photonic Bands (MPB, see ref.[8]) are shown.

the effective index of each pillar lies naturally between the refractive index of air and that of diamond (Fig. 1**b** of the main text), the pillars are inherently anti-reflective with an average transmission efficiency of 88.6%, which is higher than the 83% transmission efficiency predicted for an air/diamond interface by normal incidence Fresnel coefficients (Fig. 2**c**).

Supplementary Note 3. ELECTRON BEAM LITHOGRAPHY METHODS: PROCESS CHARACTERIZATION, DATA PREPARATION AND PROXIMITY EFFECT CORRECTION

An Elionix ELS-7500EX 50 keV electron beam lithography (EBL) tool was used to generate the metalens pattern in hydrogen silsesquioxane (HSQ), a common negative tone EBL resist, atop diamond. Using a 300 µm field size and a beam current of 1 nA on a 5 nm beam step size (shot pitch), the final pattern was exposed as a direct result of careful process characterization and modeling. In this section, we will describe the patterns and methods to generate the the proximity effect correction (PEC) parameters for the metalens.

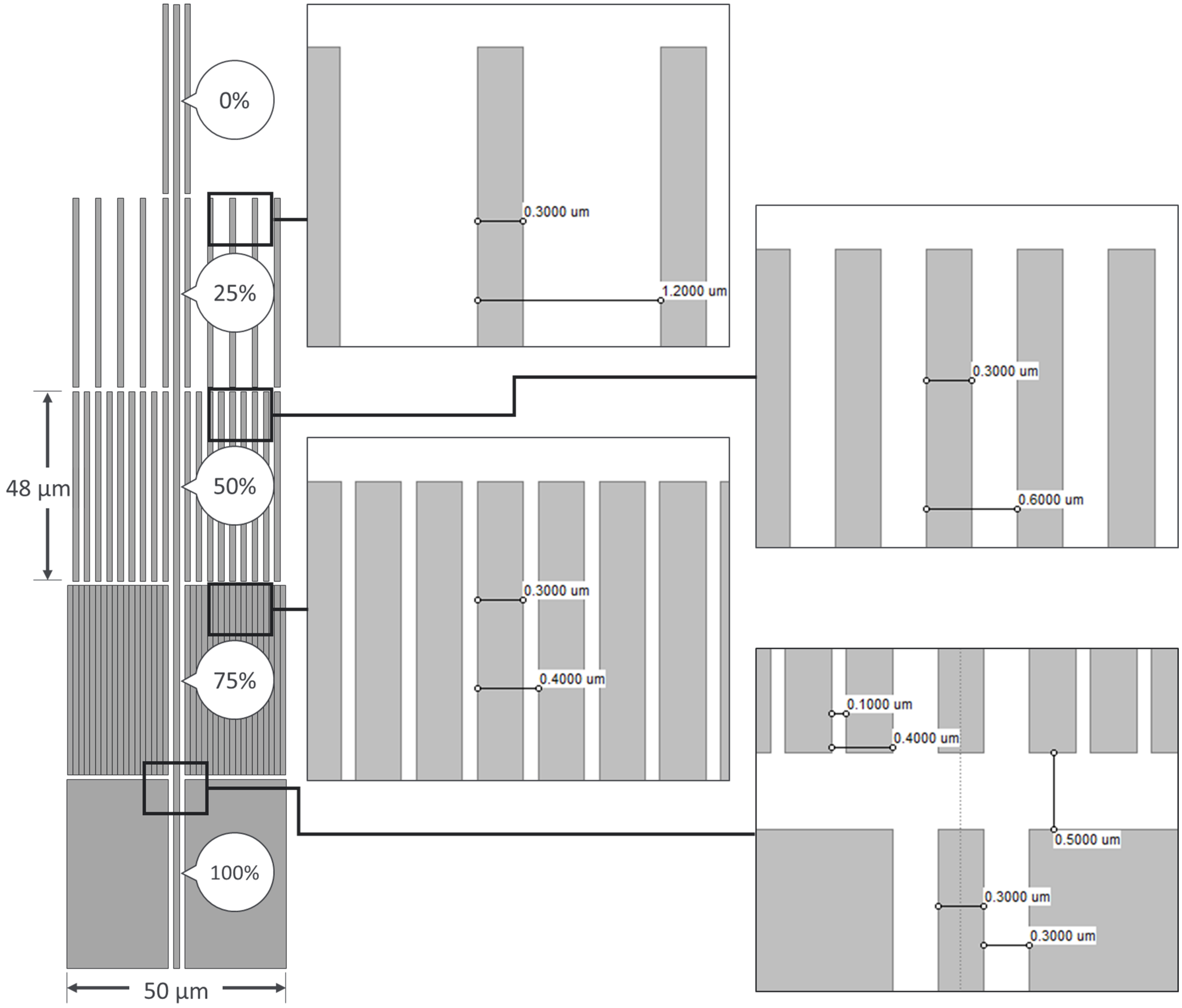

Supplementary Figure 3. **Tower Pattern.** A line-space tower pattern whose subsequent regions consist of 0%, 25%, 50%, 75% and 100% pattern densities is illustrated. The pattern density, which is referenced at the center of the pattern, is determined by the pitch of each area and whose region measures over 4β by 4β in size. The corresponding line-width and pitch per pattern density is provided. The height of each region is 48 µm tall and 50 µm wide with a vertical gap of 0.5 µm between regions. Measurements are performed in the center of each defined region.

Calibration Pattern

To calibrate the resist process, a tower pattern of lines and spaces was exposed in a dose matrix. Illustrated in Fig. 3 is the line and space tower pattern of various pitch representing

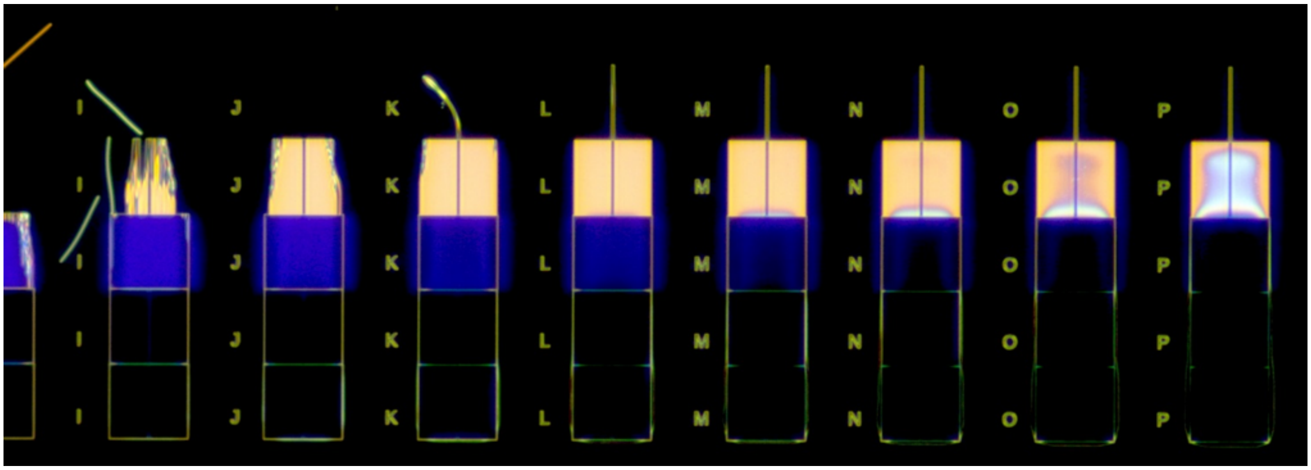

Supplementary Figure 4. **Exposure Latitude of Tower Pattern.** Tower patterns written in HSQ atop Si seen using an optical microscope in a dark field mode. Exposure latitude data by post processing scanning electron microscope images of the line widths at different pattern densities across different doses.

0%, 25%, 50%, 75% and 100% pattern densities. According to Monte Carlo simulations performed using TRACER[11] by GenISys, exposing with a 50 keV tool atop Si yields a backscatter length (β) of 10 µm. Therefore, each pattern density region is 4β by 4β or greater in size such that the center of the pattern, when exposed, has a total absorbed energy that is saturated from backscattered electrons.

A specific pattern density is achieved by applying a specific pitch to the line-space pattern. For example, a 25% pattern density consists of 300 nm lines on a 1200 nm pitch, where the line occupies 25% of the full pitch. The line-width and pitch dimensions are provided in Fig. 3. After exposure and development, the final pattern seen in Fig. 4 is imaged using a scanning electron microscope (SEM). The SEM images are post processed to extract the pattern density dependent exposure latitudes.

Process modeling and Correction

PEC is an edge-correction technology in which the absorbed energy of the resist in the pattern is analyzed and dose assignments are made such that the absorbed energy at threshold lands at the edge of the intended design. This threshold is associated with the resist sensitivity and development chemistry. Densely written patterns build up additional ab-

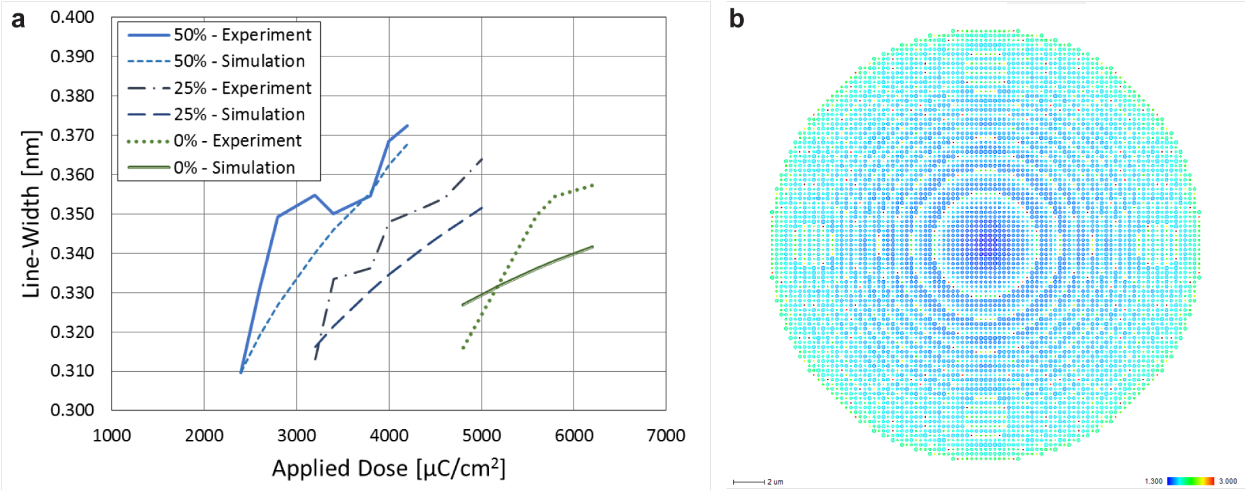

Supplementary Figure 5. **Exposure Latitude vs. Simulation.** **a**, The exposure latitude curves for 0%, 25% and 50% pattern densities. Electron beam simulation fits the empirical data with an RMS = 8 nm. **b**, Using the parameters from Tab. 2, PEC was applied to the metalens design. The colors indicate various dose factors that are necessary to print the features to size in HSQ.

sorbed energy *via* electron backscatter, requiring a local dose reduction; conversely, sparsely written (low density) patterns require an increase in local dose. The amount of background energy at these dense and sparse pattern densities directly impacts the exposure latitude, which is the critical dimension response to a change in dose.

HSQ has been shown to exhibit non-ideal behavior in its response to proximity effect correction methods due to microloading effects during resist development[12]. Using BEAMER[13] by GeniSys, a genetic algorithm was employed to model the empirical exposure latitude data. For this simulation, only the 0%, 25% and 50% pattern density data were of interest since the metalens pattern density falls within this range. Reducing the input data reduces the convergence time. The parameters used to obtain the model fit were the effective process blur, development bias, and base dose. These values are determined *via* simulation in the genetic algorithm by matching the simulated resist edge contours to the experimental exposure latitude data obtained from the tower pattern in Fig. 3 that was exposed in a dose matrix as shown in Fig. 4. The resulting effective process blur is then convolved into the electron point spread function to perform the simulation. The slope of the experimental exposure latitude data is matched in simulation by changing the effective process blur accordingly (Fig.5**a**). By adding two extra degrees of freedom, development bias and base

dose, the algorithm can converge properly.

α	5 nm
β	10 µm
Effective Blur	67 nm
Bias	-5 nm

Supplementary Table 2. Proximity Effect Correction Parameters

The final pattern was proximity effect corrected using the parameters in Table 2. As a result, the metalens is fractured such that the shapes receive the appropriate dose to print the features to size (Fig. 5**b**).

Supplementary Note 4. IMAGING WITH THE METALENS

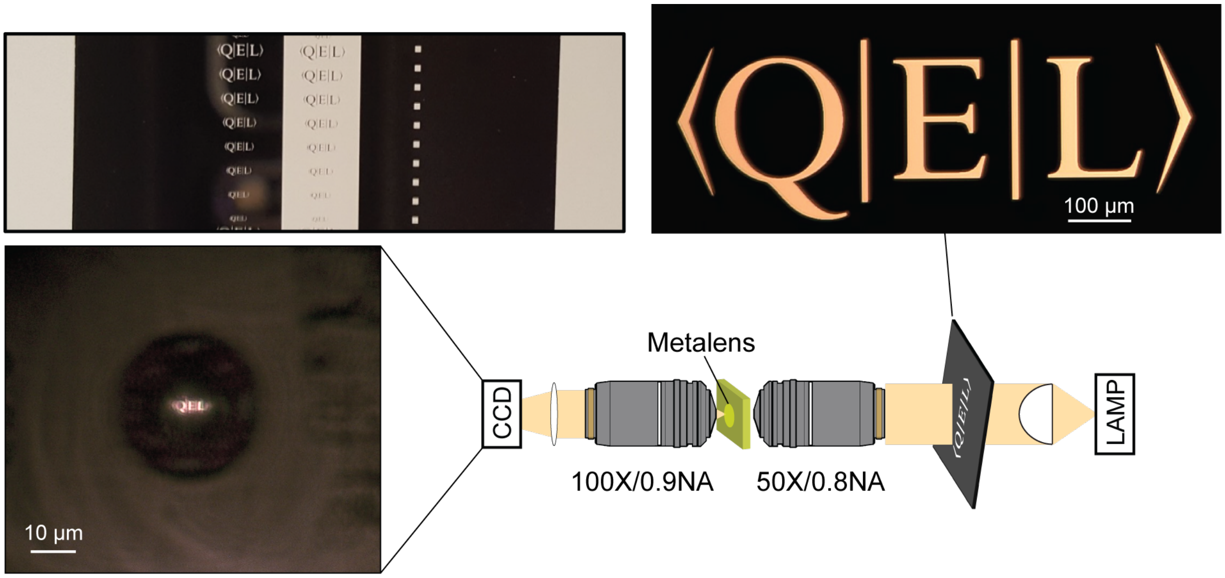

Supplementary Figure 6. **Imaging using the metalens. a**, Photograph of the chromium shadow mask fabricated on a 3"x1" glass slide. **b**, Bright-field microscope image of the shadow mask pattern imaged in (**c**) through the metalens using the setup in (**d**).

The HPHT diamond hosting the metalens is placed in a conventional upright microscope (Olympus, BX41) for brightfield transmission and reflection imaging (Fig. 2 of the main text). The bright-field transmission microscope image in Fig. 2**d** of the main text was

created by placing a chromium shadow mask between a lamp and a focusing objective, which was focused through the metalens and imaged on a CCD using a second objective as described in Fig. 6. The shadow mask was fabricated by e-beam depositing chromium on a glass microscope slide (Fig. 6**a**), and creating the pattern shown in Fig. 6**b** with a combination of photolithography and chemical etching. The resulting CCD image shown in Fig. 6**c** was created using the transmission microscope shown in Fig. 6**d**.

Supplementary Note 5. METALENS CHARACTERIZATION

Measurement setup

The diamond is mounted on a glass cover slip, which is secured to the stage of a custom-built laser-scanning confocal microscope (Fig. 7) for characterization and NV center imaging (Figs. 3,4 of the main text). The laser-scanning confocal microscope has two optical paths for simultaneously probing the metalens from air and through the diamond substrate: a fiber-coupled path and an objective path. The objective path consists of a $4f$ relay-lens system with achromatic doublet lenses (L3 and L4, Newport, 25.4 mm × 150 mm focal length, PAC058AR.14), which is used to align the back aperture of the objective to a fast-steering mirror (FSM, Optics in motion, OIM101). This is followed by a 560 nm long-pass dichroic mirror (Semrock, BrightLine FF560-FDi01) which directs the 532 nm excitation laser (Coherent, Compass 315M-150) into the objective (OL, Nikon, Plan Flour x100/0.5-1.3) while wavelengths above 560 nm are passed through a 532 nm and a 568 nm long-pass filter (Semrock, EdgeBasic BLP01-532R, EdgeBasic BLP01-568R) before being focused down to a 25 µm-core, 0.1 NA, multimode fiber (Thorlabs M67L01) *via* the achromatic doublet lens (L5, Newport, 25.4 mm × 50 mm focal length, PAC049AR.14). The multimode fiber is then connected to a single-photon counting module, (SPCM, Excelitas, SPCM-AQRH-14-FC) or a spectrometer (Princeton Instruments, IsoPlane-160, 750 nm blaze wavelength with 1200 G/mm) with a thermoelectrically-cooled CCD (Princeton Instruments PIXIS 100BX). The electrical output of the single-photon counting module is routed via BNC cables to either a data acquisition card (DAQ, National Instruments PCIe-6323) or a time-correlated single-photon counting card (PicoQuant, PicoHarp 300).

The fiber-coupled path is modified to enable different experiments conducted on the

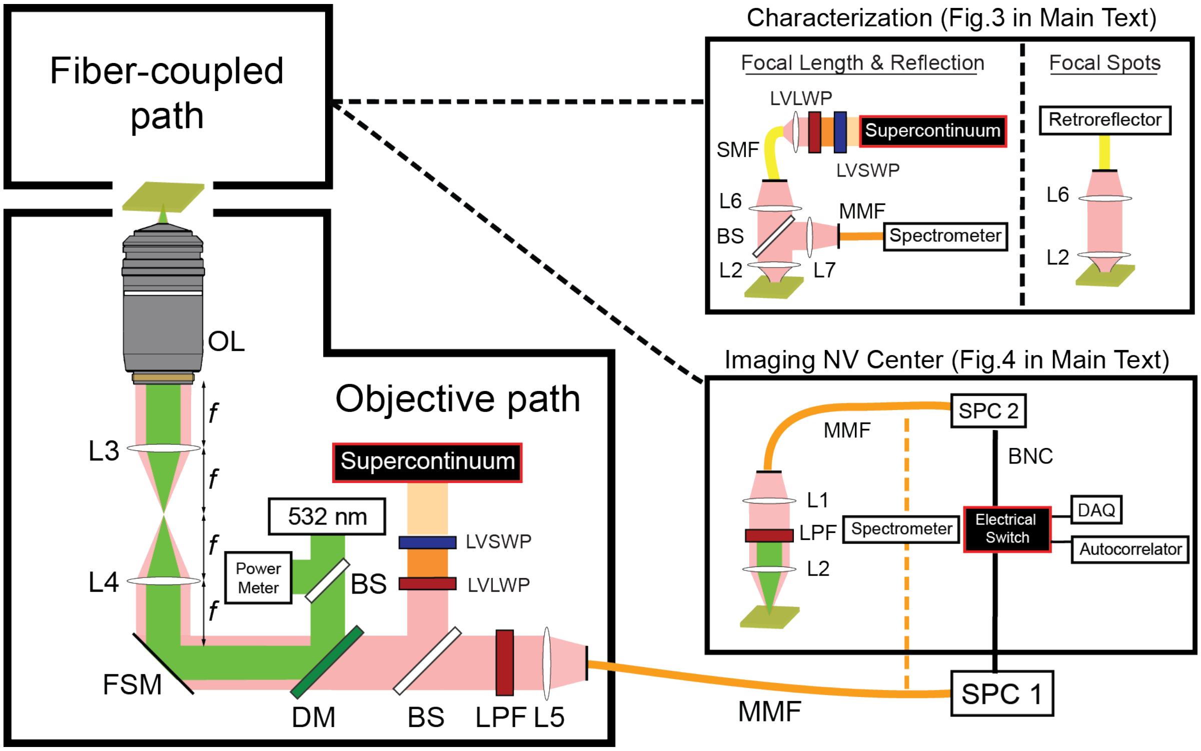

Supplementary Figure 7. **Metalens Characterization and Imaging setup.** Experimental setup for characterization of the metalens and imaging an NV center through a confocal microscope. The setup is divided into the fiber-coupled path - where the metalens is coupled to either a single- or multi-mode fiber - and the objective path which enables confocal excitation and collection through an oil-immersion objective. The fiber-coupled path is modified to allow different experiment configurations with the metalens, as shown in the figure. The details of the full setup are discussed in the text.

metalens. For characterization, a broadband supercontinuum source (Fianium WhiteLase SC400) was brought into the objective path via a beamsplitter cube (Thorlabs BS014). A $f = 2.0\,\mathrm{mm}$ collimating lens (L6, Thorlabs CFC-2X-A) and a $f = 15\,\mathrm{mm}$ achromatic lens (L2, Thorlabs AC064-015-B) were used to couple the metalens to a fiber retroreflector (Thorlabs S1TM09) which, upon reflection, recreates a 28 µm diameter Gaussian beam that emulates the planewave source used in our FDTD simulations. The excitation wavelength is set by passing the supercontinuum beam through a set of linear variable short-pass (Delta Optical Thin Film, LF102474) and long-pass filters (Delta Optical Thin Film LF102475) prior to fiber-coupling, which can be adjusted to filter out a single wavelength with $< 8\,\mathrm{nm}$ bandwidth or be removed completely for broadband excitation. For reflectance and focal

lengths measurements, the supercontinuum source is coupled instead to lens L6, and a beamspliter cube (Thorlabs, BS014) was added between the collimating and focusing lenses so that reflected light could be focused into a 200 µm-core MMF (Thorlabs, M25L01) that is coupled to a spectrometer (Thorlabs CCS100) using a $f = 100$ mm achromatic doublet lens (L7, Newport, PAC052AR.14). To modify this setup for imaging an NV center, a 532 nm and a 568 nm long-pass filter (Semrock, EdgeBasic BLP01-532R, EdgeBasic BLP01-568R) is placed after L2 and the filtered light is focused down to a 25 µm-core, 0.1 NA, multimode fiber (Thorlabs M67L02) with a $f = 13$ mm achromatic doublet lens (L1, Thorlabs, AC064-013-B). The multimode fiber can then be connected to a single-photon counting module or a spectrometer as described in the previous paragraph.

Calibration

Calibration of the fast-steering mirror (FSM) is critical for characterization of the metalens's point-spread function at focus. To perform this calibration, a PL scan of the metalens surface was taken with 532 nm pump beam (Fig. 8**a**), and the image was compared to the CAD layout of the metalens pattern (Fig. 8**b**) to determine the differential voltage required to move the FSM by a known distance in x_{FSM} and y_{FSM}.

The relative shift in axial position of the confocal collection volume caused by piezo stage movements is scaled by a factor ranging from $\frac{n_{\mathrm{D}}}{n_{\mathrm{oil}}}$ to $\frac{n_{\mathrm{D}} \cos\theta_{\mathrm{D}}}{n_{\mathrm{oil}} \cos\theta_{\mathrm{oil}}}$, where $\theta_{\mathrm{D,oil}} = \sin^{-1}\left(\frac{\mathrm{NA}}{n_{\mathrm{oil,diamond}}}\right)$ are the maximum focusing angles in diamond and oil, respectively[14]. We calculate this scaling factor using our numerical PSF model described in the methods section, and find that it is $\approx \frac{n_{\mathrm{D}}}{n_{\mathrm{oil}}}$ (Fig. 8**c**), which is applied to the measured piezo stage position, z'_{piezo}, to find the physical displacement of the confocal volume within the sample, $z_{\mathrm{piezo}} \approx \frac{n_{\mathrm{D}}}{n_{\mathrm{oil}}} z'_{\mathrm{piezo}}$. The dispersive refractive index of diamond, $n_{\mathrm{D}}(\lambda)$, used for the calculations in Fig. 8**c** was modeled using the Sellmeier equation with coefficients from ref.[15]. The sample thickness was checked by focusing 532 nm on both the bottom surface and top surface of the diamond, and measuring the relative position on the piezo stage. The piezo stage displacement was 92 µm, and the iris of the objective was set to $\mathrm{NA}_{\mathrm{obj}} = 0.5$. The numerically calculated scaling factor 1.6, giving a sample thickness of 147 µm.

The objective lens used has an adjustable iris, which effectively reduces the NA to mitigate spherical aberration. The collar was set to $\mathrm{NA}_{\mathrm{obj}} \approx 0.75$, which was confirmed by

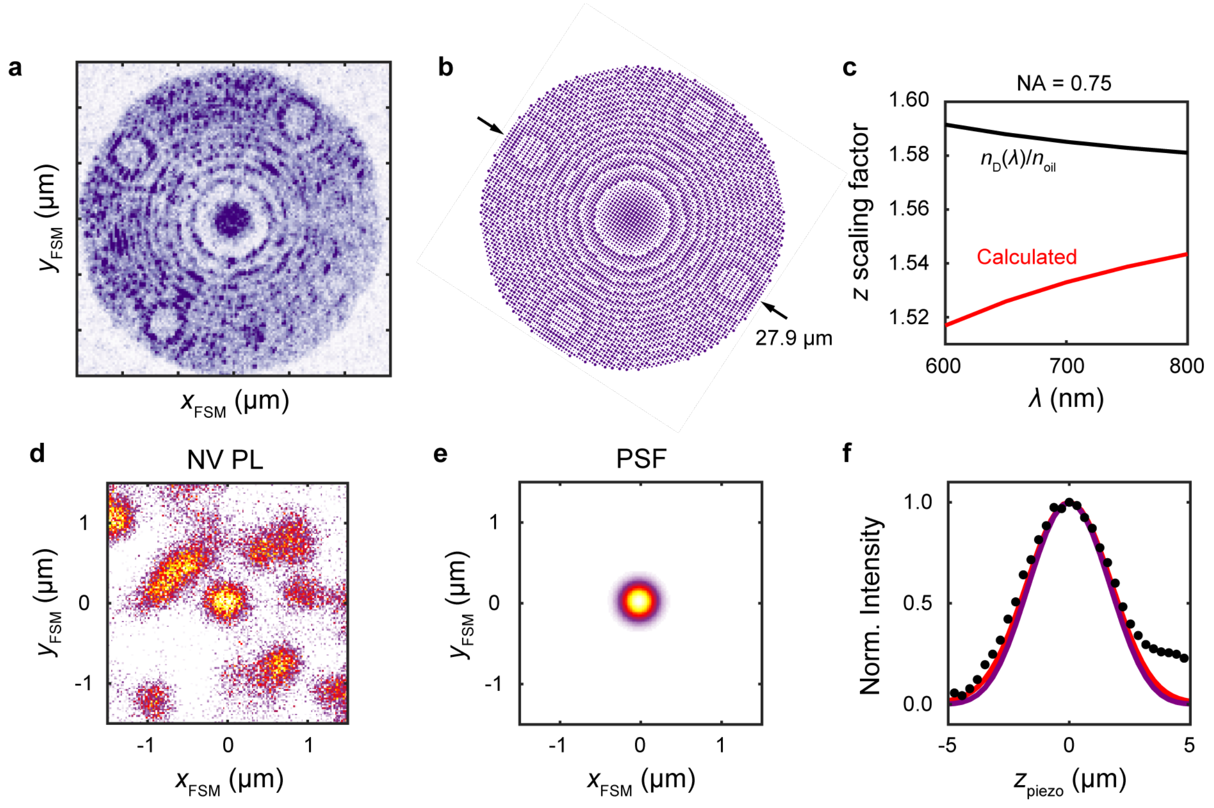

Supplementary Figure 8. **Scanning microscope calibration.** **a**, PL scan of metalens surface, used to calibrate the fast-steering mirror (FSM). **b**, CAD layout of pillars. **c**, Objective focus position shift with $\hat{z}$-piezo stage movement. **d**, PL scan through the confocal path with the $\hat{z}$-piezo stage positioned at the metalens focus. **e**, Fit to an isolated NV, used to determine $\mathrm{NA_{obj}} \approx 0.75$. **f**, Axial scan of an isolated NV (black circles), ideal axial response (purple curve) and calculated axial response with spherical aberration (red curve).

measuring PL from an isolated NV center (Fig. 8**d**). The image formed by a laser scanning confocal measurement of an isolated quantum emitter can be approximated as the incoherent product of the paraxial microscope intensity PSF at pump and PL wavelengths[16], $I \approx |I_0(\lambda_{\mathrm{pump}})|^2 \cdot |I_0((\lambda_{\mathrm{PL}})|^2$, where I_0 is the lowest-order diffraction integral. In the transverse plane this diffraction integral results in an Airy disk, $I_0 = \frac{2J_1\left(\mathrm{NA_{obj}}k_0(r_{\mathrm{image}}-r_0)\right)}{\mathrm{NA_{obj}}k_0(r_{\mathrm{image}}-r_0)}$ with $(r_{\mathrm{image}} - r_0) = \sqrt{(x_{\mathrm{FSM}} - x_0)^2 + (y_{\mathrm{FSM}} - y_0)^2}$, $k_0 = 2\pi/\lambda$, and $\mathrm{NA_{obj}}$ is the numerical aperture of the imaging objective. We found that the PSF of our microscope was not limited by the spot size of the excitation beam (either due to operating at saturation, or the pump beam not being diffraction limited), as such the PSF at the pump wavelength was replaced

with the collection PSF at PL wavelength, $I = |I_0(\lambda_{\mathrm{PL}})|^2 \cdot |I_0(\lambda_{\mathrm{PL}})|^2$. This equation is used to fit the NV measurement in Fig. 8**d** with $\mathrm{NA_{obj}}$ as a free parameter; the results are shown in Fig. 8**e**. Fits were performed using both $\lambda_{\mathrm{PL}} = 700\,\mathrm{nm}$ and a weighted fit over the NV PL spectrum, resulting in fit values of $\mathrm{NA_{obj}} = 0.76 \pm 0.03$ and $\mathrm{NA_{obj}} = 0.73 \pm 0.03$, respectively.

An axial measurement of an isolated quantum emitter taken by scanning the piezo stage is described by[16] $I(x_{\mathrm{FSM}} = 0, y_{\mathrm{FSM}} = 0, z_{\mathrm{piezo}}) = \left|\mathrm{sinc}\left(\frac{\mathrm{NA}^2_{\mathrm{obj}} z_{\mathrm{piezo}}}{2n^2_{\mathrm{oil}}\lambda}\right)\right|^4$, where $n_{\mathrm{oil}} = 1.518$ is the refractive index of the immersion oil used with our objective. Using the fit value of $\mathrm{NA_{obj}} \approx 0.75$, the axial PSF (red curve) is compared to measurements (black circles) in Fig. 8**f**.

Metalens focal length

The focal length of the metalens shown in Fig. 3**e** of the main text (right axis) was measured at five wavelengths by setting the FSM position to the peak of the transverse focused spot and scanning the piezo sample stage in the $\hat{z}$ (axial)-direction by 200 nm steps, corresponding to shifts of $\frac{n_{\mathrm{D}}}{n_{\mathrm{oil}}} \cdot 200\,\mathrm{nm} \approx 315\,\mathrm{nm}$ inside the diamond. The position of focus was determined by fitting the peak signal of the piezo scan at each wavelength to a Gaussian (Fig. 9**a**). The bright PL of the metalens surface was also fit with a Gaussian (Fig. 9**b**) and used to calibrate the relative distance between the sample surface and the metalens focus.

The chromatic aberration of our imaging system was checked by feeding the supercontinuum through the collection line and measuring the location of the metalens's surface *via* a CCD camera in the collection path. Since the supercontinuum is coupled to a SMF, we can achieve this by simply coupling the SMF to the MMF in the objective collection path with an FC-to-FC fiber connector (Thorlabs, ADAFC1). By verifying that the surface location is the same when the excitation source is band-passed to 600 nm as when it is 800 nm, the chromatic aberration of the system was found to be negligible.

Metalens focal spot

To make an accurate comparison between the 3D-FDTD simulated metalens focal spot, $I_{\mathrm{ML}} = |\vec{E}_{\mathrm{ML}}|^2$ (Fig. 3**d** of the main text, shown at five wavelengths in Fig. 10**a**), and our measurements, distortions in the measured image caused by aberrations and the finite resolution

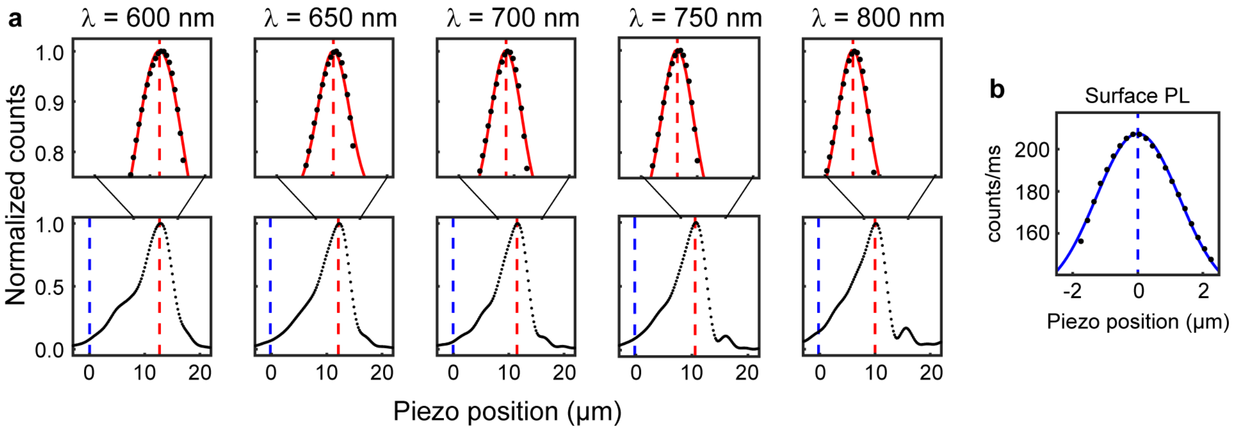

Supplementary Figure 9. **Focal length measurements.** **a**, Normalized signal versus piezo stage position for five wavelengths. **b**, Surface PL used to locate the sample surface.

of our microscope need to be taken into account. The metalens focal spot measurements described in the methods section produce the images shown in Fig. 10**d**, which represent the number of photons collected by the SPCM at a position in the sample, $\vec{r}_{\text{image}}$, defined by the FSM in the transverse directions and by the sample stage in the axial direction: $\vec{r}_{\text{image}} = x_{\text{FSM}} \cdot \hat{x} + y_{\text{FSM}} \cdot \hat{y} + z_{\text{piezo}} \cdot \hat{z}$. To account for scattering and background subtraction, the same measurements are repeated with the surface of the sample covered by absorptive tape, blocking the light transmitted through the metalens from entering the top collection optics shown in Fig. 7. For transverse scans, the background corrected image is an incoherent convolution[17] of the microscope intensity PSF with the electric field amplitude of the metalens focal spot, $|\vec{E}_{\text{ML}} * \text{PSF}|^2$. We model the PSF of our microscope (Fig. 10**b**) by numerically evaluating the diffraction integrals, I_0, I_1, I_2, that define the dyadic Green's function of a high-NA optical system[16] with the inclusion of an aberration function that accounts for the optical pathlength difference introduced by imaging through a media with mismatched refractive indices[18] ($n_{\text{oil}} = 1.518$ and $n_{\text{D}} = 2.4$ for our measurement setup). A value of $\text{NA}_{\text{obj}} = 0.76 \pm 0.03$ was used in these calculations, corresponding to the position of the adjustable iris on our Nikon 100x objective, which was confirmed by fits to NV PL scans as described in Supplementary Note 5.

A convolution of the simulated metalens focal spot from Fig. 10**a** with the calculated PSF from Fig. 10**b** is shown in Fig. 10**c**, which exhibits remarkable agreement with the measurements in Fig. 10**d**. In order to compare these measurements directly to the sim-

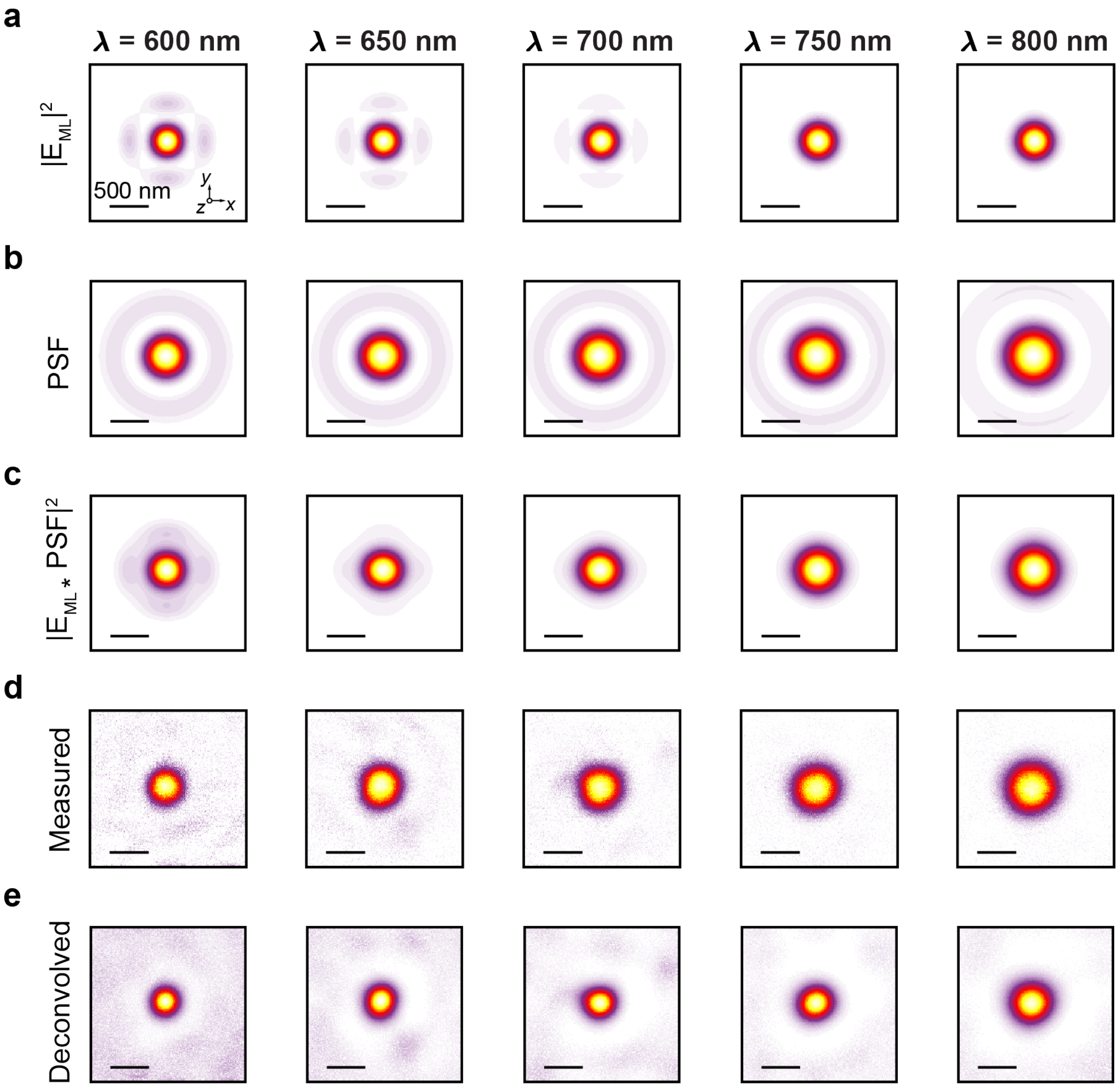

Supplementary Figure 10. **Measurement and simulation of the metalens focal spot at five wavelengths.** **a**, 3D-FDTD-simulations of the metalens focal spot. **b**, Calculated point spread function (PSF) of the Nikon 100x oil immersion objective used for the measurements described in Fig. 4 of the main text with spherical aberration caused by imaging through diamond included. The value of the NA used in these calculations was determined by fits to the spatial maps of NV PL shown in Fig. 8**d-f**. **c**, Incoherent convolution of the simulated metalens focal spot (**a**) with microscope point-spread function (**b**). **d**, Metalens focal spot measured by incoherent imaging. **e**, Measured focal spot (**d**) after deconvolution of the PSF (**b**). All scale bars correspond to 500 nm

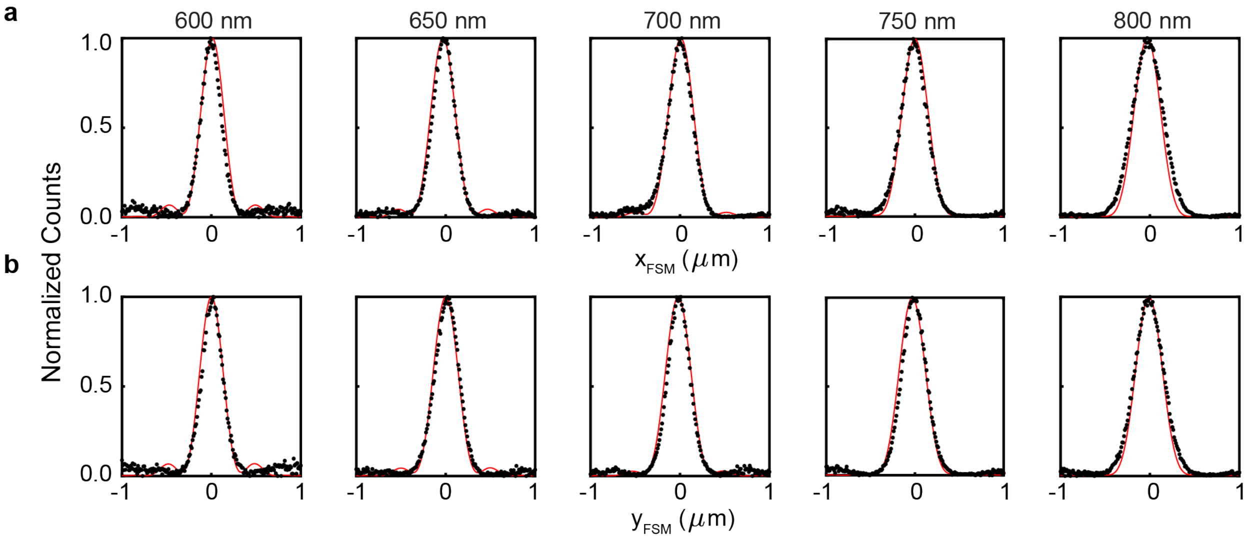

Supplementary Figure 11. **Comparison of measured and simulated transverse cross-sections of the metalens focus spot at five wavelengths. a,b,** Transverse cross-sections of FDTD simulated focus spot (red curves) and measured focus spot with objective point-spread function deconvolved (black points).

ulated focal spot in Fig. 10**a**, the PSF from Fig. 10**b** is deconvolved from Fig. 10**d** using the Lucy-Richardson method implemented in MATLAB's **deconvlucy** function with the number of iterations chosen by minimizing the mean-squared-errors of the deconvolution, resulting in the images shown in Fig. 10**e**. Comparisons of x and y cross-sections of the simulated metalens focal spot (Fig. 10**a**) and deconvolved measurements (Fig. 10**e**) are shown in Fig. 11**a,b**, respectively.

Focusing in air

To measure the focus spot formed in air when the metalens is illuminated by a collimated beam from inside of the diamond (Fig. 12**a**), the diamond substrate is mounted upside-down on the inverted microscope shown in Fig. 7 with the metalens facing downwards towards a 100x air objective (Olympus, UMPlanFl 100×/0.90) in the objective path. A 633 nm He-Ne laser source (Melles Griot 05-LHP-153) is SMF-coupled and collimated *via* a $f = 2.0\,\mathrm{mm}$ collimating lens (Thorlabs CFC-2X-A) to illuminate the back-side of the diamond substrate from the fiber-coupled path. The procedure for measuring the focus spots and focal length

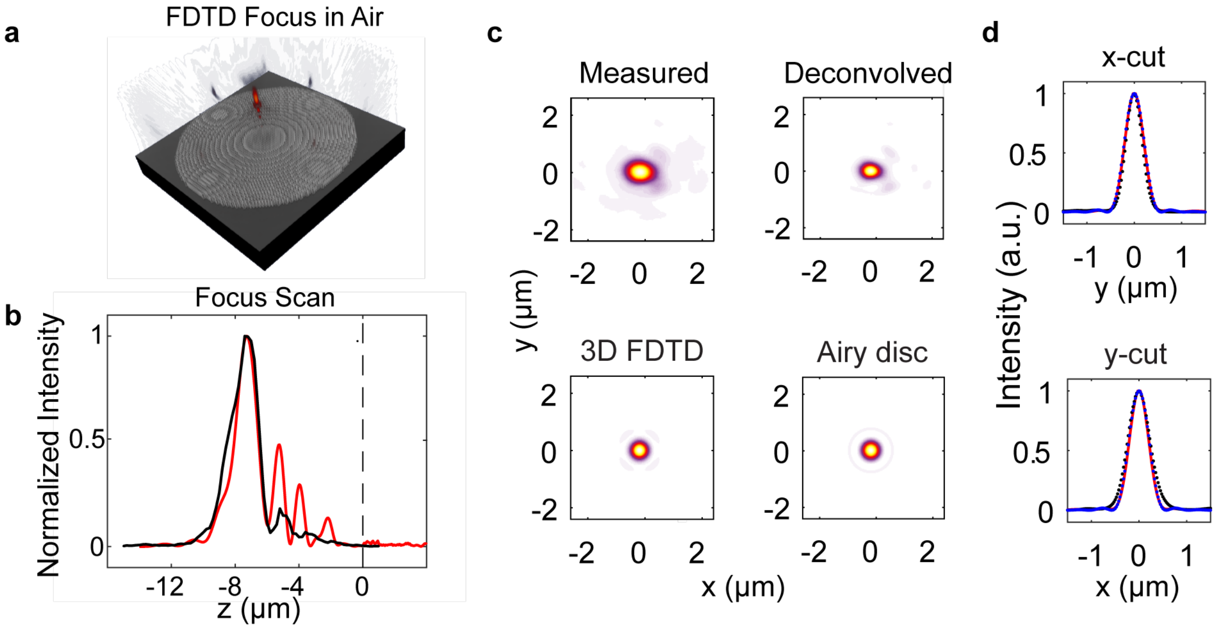

Supplementary Figure 12. **Simulated and Characterized Focus in Air.** **a**, FDTD simulation of the metalens focus in the air. **b**, FDTD (red) and measured (black) focus scan with the FSM at the center of the metalens. Dash line at $z = 0$ denotes surface of the metalens. The objective focus is moved further away from the metalens as z becomes more negative. **c**, Measured focus spot plotted alongside deconvolved, FDTD, and airy disc focus spots. **d**, $x-$ and $y-$axis cross-section at focus of the deconvolved spot (black), the airy disc (blue), and FDTD simulation (red).

of the metalens are described in the Experimental section of Methods, as the collection path after the objective is identical to the objective path shown in Fig. 7. An axial scan of the metalens focus in air is plotted in Fig. 12**b**, showing an excellent agreement with the FDTD simulation. The measured transverse focus spot, shown in Fig. 12**c**, is deconvolved using blind deconvolution with MATLAB's built-in **deconvblind** function. The cross-sections of the deconvolved focus spot, plotted in Fig. 12**d** in black, demonstrate again an excellent agreement with the FDTD simulation (red).

Supplementary Note 6. TRANSMISSION, COLLECTION, AND FOCUSING EFFICIENCIES

The purpose of our metalens is to collect and collimate photons originating from a quantum emitter inside the diamond. The simulated coupling efficiency as a function of the

NA of the external collection optics (e.g., an optical fiber) is shown in Fig. 3b of the main text. Fig. 13**b** shows the corresponding angular distribution of the metalens output, which is highly concentrated within a small NA. The overall coupling efficiency accounts for this collimation along with any losses due to reflection or scattering into other optical modes. Our measurements and simulations indicate that losses in coupling efficiency are primarily due to forwarding scattering, as the metalens surface has a high transmission efficiency.

The low reflectivity seen qualitatively in Fig. 2**c** of the main text is quantified by simulations and measurements to be below 12% (Fig. 13**a**). The simulated reflection spectrum in Fig. 13**a** was calculated by integrating the time-averaged Poynting vector, $S_z = -\frac{1}{2}\mathrm{Re}\left\{\vec{E} \times \vec{H}^*\right\} \cdot \hat{z}$, over a 30 µm × 30 µm surface, 0.1 µm above and 0.4 µm below the metalens within the TFSF source volume. The simulation volume was reduced to 31 µm × 31 µm × 2 µm and the number of wavelength points was increased to 41 for these simulations.

For reflection measurements (Fig. 13**a**) a $f = 15$ mm achromatic lens (Thorlabs AC064-015-B) is used to focus the collimated excitation beam to a $\sim$ 30 µm-diameter spot at the top surface of the diamond. A beamspliter cube (Thorlabs BS014) was added between the collimating and focusing lenses so that reflected light could be focused into a 200 µm-core MMF (Thorlabs, M25L01) that is coupled to a spectrometer (Thorlabs CCS100) using a $f = 100$ mm achromatic doublet lens (Newport, PAC052AR.14). The reflectance spectrum in Fig. 13**a** was normalized using measurements of the reflected optical power measured with the fiber-coupled path aligned to the metalens, $P_{\mathrm{ML}}(\lambda)$, and off the metalens on a planar region of the diamond surface, $P_{\mathrm{surface}}(\lambda)$, using the following expression:

$$R_{\mathrm{ML}}(\lambda) = \frac{P_{\mathrm{ML}}(\lambda)}{P_{\mathrm{surface}}(\lambda)} R_{\mathrm{surface}}, \tag{S2}$$

where $R_{\mathrm{surface}} = \frac{P_{\mathrm{surface}}(\lambda)}{P_{\mathrm{in}}(\lambda)}$ is the reflectance of an air/diamond interface and is calculated using Fresnel coefficients to be 17% at normal incidence. The ripples in Fig. 13**a** are due to ghosting from the beam splitter cube used to collect the reflected signal. The measured reflectance spectrum is slightly lower than the simulated spectrum (both plotted in Fig. 13**a**). The source of the discrepancy is believed to be due to the NA of our top collection optics. The simulations represent the reflected light over all angles (specular and scattered), while our collection optics only cover a limited range of angles.

To confirm that the NA of our metalens found by fitting deconvolved measurement data

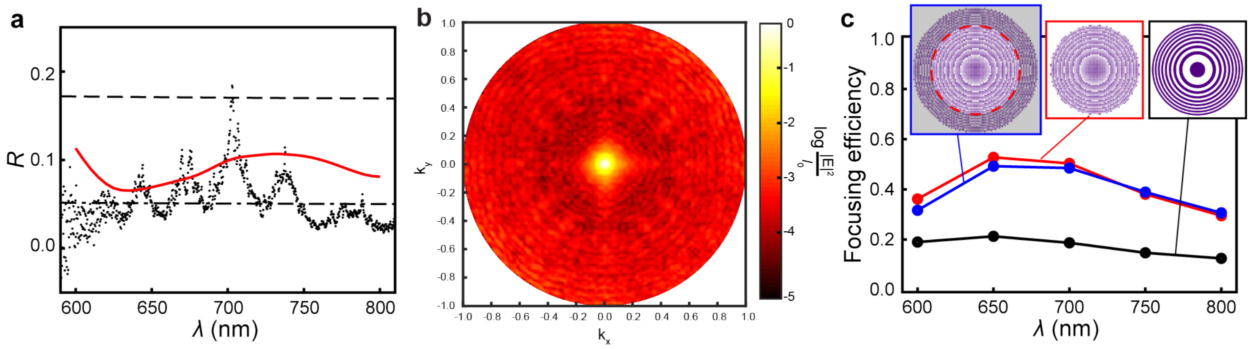

Supplementary Figure 13. **Transmission, collection, and focusing efficiencies. a** Simulated (red curve) and measured (points) metalens reflectivity. The dashed lines indicates the ∼17% and ∼5% reflectivities of planar air/diamond and immersion oil/diamond interfaces. **b**, Calculated momentum distribution of the collimated metalens output in air from an NV center at focus. **c**. Simulated focusing efficiency of the 27.9 µm-diameter fabricated metalens (blue), the same metalens with a decreased physical diameter equal to the 19.3 µm effective aperture of our fabricated metalens (red), and a 19.2 µm-diameter binary Fresnel zone-plate (black).

shown in Fig. 3 of the main text corresponded to an effective aperture that is smaller than the physical diameter of the metalens, we also performed 3D-FDTD simulations of the focusing efficiency shown in Fig. 13**c**. These simulations were performed using a total-field/scattered-field (TFSF) planewave source as described in the Methods section, and the focusing efficiency represents transmission at the metalens focus, calculated by integrating the z-component of the Poynting vector over a 3 µm × 3 µm box at focus. The blue curve in Fig. 13**c** shows the focusing efficiency of the fabricated metalens pattern (inset, blue border) when normalized to the power contained in $\pi(\frac{19.3\,\mu\mathrm{m}}{2})^2$ area of the incident plane wave (inset, red, dashed circle), while the red curve shows the focusing efficiency of a metalens pattern with a diameter equal to the 19.3 µm effective aperture (inset, red border). The agreement between these two curves illustrates the need for an alternate metalens design approach for wide angles. The focusing efficiency of our metalens peaks at $\sim 50\%$, which is consistent with other demonstrations of high-NA metalenses[19,20], and far exceeds the focusing efficiency of an equivalent dielectric Fresnel zone plate (Fig. 13**c**). The dielectric zone plate was designed with a fixed height of $\lambda/n = 700\,\mathrm{nm}/2.4 = 292\,\mathrm{nm}$ such that each zone produces a binary 2π transmission phase mask[21].

We can use measurements of the saturated photon count rate (Fig. 4e in the main text)

to estimate the metalens photon collection efficiency for comparison with these calculations. The collection efficiency, spectrally averaged over the NV center's PL band, is given by

$$\langle \eta_{\text{tot}} \rangle_\lambda = \frac{S^{\text{sat}}_{\text{SPCM}}}{S^{\text{sat}}_0} \tag{S3}$$

where S^{sat}_0 is the NV center's intrinsic photon emission rate at saturation, and $S^{\text{sat}}_{\text{SPCM}}$ is the observed saturation count rate in our single-photon counting module (SPCM). The total collection efficiency can be deconstructed to $\eta_{\text{tot}} = \eta_{\text{geom}} \cdot \eta_{\text{coupling}} \cdot \eta_{\text{optics}} \cdot \eta_{\text{SPCM}}$, comprising of the geometrical efficiency of the metalens, η_{geom}, the coupling efficiency of the metalens, η_{coupling}, the transmission efficiency of our collection optics, η_{optics}, and η_{SPCM}, the quantum efficiency of our SPCM. The spectrally averaged coupling efficiency, $\langle \eta_{\text{coupling}} \rangle_\lambda$, shown in Fig. 3**b** of the main text is calculated from simulations of the angular distribution of the collimated metalens output in air (Fig. 13**b**) as described in the Methods section.

The geometrical efficiency is determined by the NA of the metalens from the following equation[22]:

$$\eta_{\text{geom}} = \frac{1}{8}\left\{4 - 3\cos\theta_{\text{max}} - (\cos\theta_{\text{max}})^3 + 3\left[(\cos\theta_{\text{max}})^3 - \cos\theta_{\text{max}}\right]\cos^2\Delta_{\text{em}}\right\}, \tag{S4}$$

where $\theta_{\text{max}} = \sin^{-1}\left(\frac{\text{NA}}{n_{\text{D}}}\right)$, $\Delta_{\text{em}} = 90°, 35.3°$ are the angles between the NV dipole moments D_x, D_y and the optical axis of the objective/metalens. Averaging over both dipoles and the NV emission spectrum, we calculate $\langle \eta_{\text{geom}} \rangle_\lambda = 6.82\%$. The SPCM efficiency is specified by the manufacturer to be $\eta_{\text{SPCM}} = 70\%$ We measure $\eta_{\text{optics}} = 24\%$ using the same collection optics as described in Fig. 7 for measuring an NV. To simulate the collimated output from the metalens, a 30 µm pinhole was fabricated onto a chrome mask and illuminated by the collimated supercontinuum source without the objective in the objective path (Fig. 7).

Using these values, we can estimate the spectrally averaged coupling efficiency, $\langle \eta_{\text{coupling}} \rangle_\lambda$, given the saturated count rate of $S^{\text{sat}}_{\text{SPCM}} = 87.3$ kCts/s and a corresponding value for S^{sat}_0. The NV center's response to optical saturation is nontrivial due to the combined effects of shelving in the singlet state and ionization/recombination transitions that rapidly cycle between the neutral and negative charge states. At very high optical intensity, these effects actually cause the observed emission rate to drop as the excitation rate is further increased. That nonmonotonic regime is not reached in our experiments, although the count rate clearly saturates in Fig. 4**e** of the main text. Estimates for the corresponding maximum

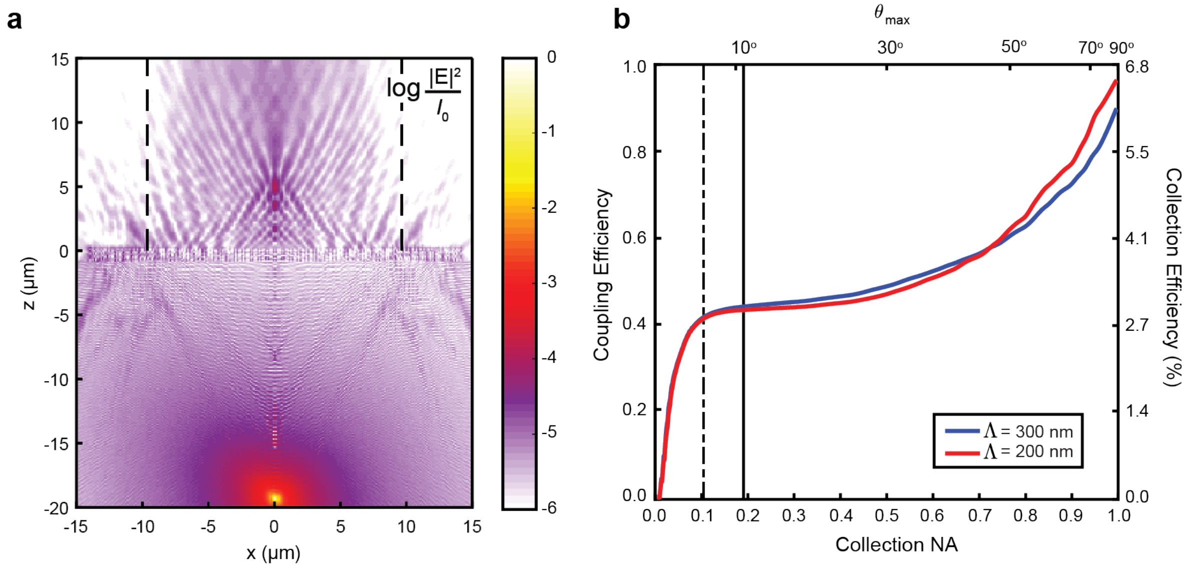

Supplementary Figure 14. **Simulating a reduced-pitch design.** **a**, Steady-state field intensity simulated for a metalens of reduced lattice constant (200 nm). The vertical dashed lines correspon to the effective aperture of the original design. **b**, Simulated coupling efficiency as a function of coupling fiber NA for two metalenses designed with different lattice constants: the original metalens studied throughout the text with a pitch of 300 nm (blue) and a second metalens with a pitch of 200 nm designed below the diffraction limit in diamond (red). The acceptance angle corresponding to NA is plotted as a second x-axis. Dot-dashed and solid black lines indicate NA values of 0.1 and 0.19, corresponding to the NA of the optical fiber and collimating lens used in the measurements, respectively.

intrinsic emission rate, S_0^{sat}, range between 10-20 MHz depending on both shelving and charge transition rates[3,23,24].

Accounting for this uncertainty, we ultimately arrive at an estimate for the spectral averaged coupling efficiency of the metalens in the range $\langle\eta_{\mathrm{coupling}}\rangle_\lambda = $ 38-76%. This compares favorably with the calculated value of 34.8% (see Fig. 3**b** in the main text).

In prior sections, we discuss how the effective diameter of the metalens is significantly smaller than its actual diameter, as evidenced by the steady-state field intensity plotted in Fig. 3**a** in the main text. To further illustrate the need for more advanced designs and to more carefully consider diffraction as a possible limiting factor to our device's performance, we investigated the impact of our design pitch on the coupling efficiency. To eliminate first-order diffraction within the diamond, the periodicity is limited to $\Lambda < \frac{\lambda}{1+n_d} \approx 206\,\mathrm{nm}$ given

$\lambda = 700$ nm and $n_d = 2.4$. Since our design with $\Lambda = 300$ nm breaks this condition, we designed a second metalens satisfying the same Fresnel profile, but with a pillar pitch of 200 nm. We performed the same 3D-FDTD simulations done to generate Fig. 3**b** in the main text on this new design, and the results are shown in Fig. 14. The coupling efficiencies (Fig. 14**b**) are normalized by the same geometrical efficiency described in Eq. S4, using for consistency the same effective NA of the original metalens. While the reduced pitch design provides a slight improvement in total transmission — evidenced by a $\approx 6.5\%$ higher coupling efficiency for NA $= 1$ in Fig. 14**b** — this is due to increased forward scattering into large angles that does not improve coupling into low-NA collection optics. We conclude that diffraction inside the diamond was not a limiting factor in the performance of our device.

Supplementary Note 7. BACKGROUND SUBTRACTION FOR NV MEASUREMENTS

Spectra and Saturation curves

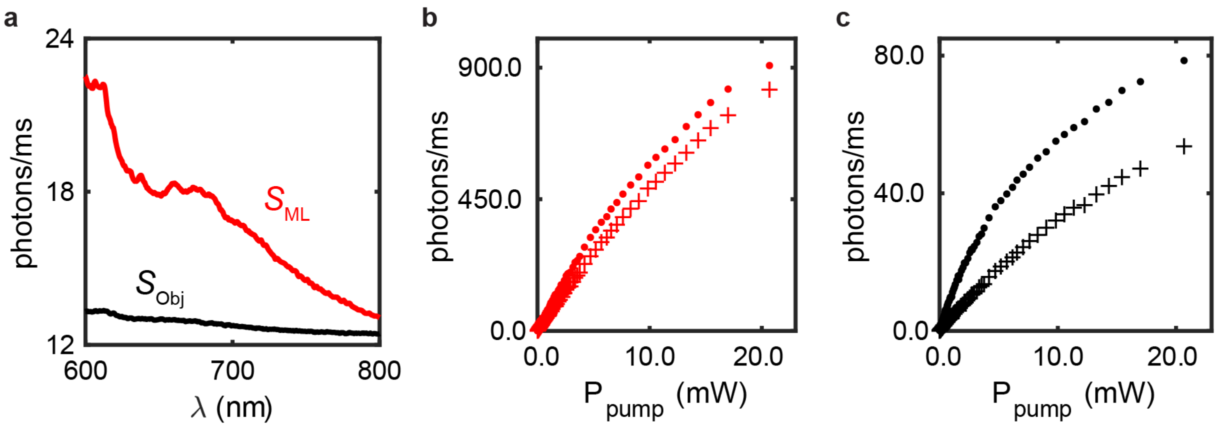

Supplementary Figure 15. **Background PL.** Measurements of background PL for correcting (**a**) NV spectra and (**b,c**) NV saturation curves in Fig. 4**d,e** of the main text. **a**, Background spectra of NV measurements, S_{ML} and S_{obj} denote signal from the metalens and objective paths, respectively. **b**, Saturation count rates signal (red solid dots) and background (red crosses) from the metalens path. **c**, Saturation count rates signal (black solid dots) and background (black crosses) from the objective path.

Experimental setup for spectra and saturation curves measurements are described in

Fig. 7. Background spectra and saturation curves are measured at a transverse scan position that is away from the NV center but still within the field-of-view of the metalens. Signal (on-NV) and background (off-NV) spectra for both metalens and objective paths were collected with a 5-min acquisition time. The background spectra for both paths, plotted in Fig. 15**a**, are subtracted from the signal spectra to yield the points plotted in Fig. 4**d** in the main text. For saturation curves, the 532 nm pump beam is passed through a variable optical-density filter (Thorlabs, NDC-50C-4), before going through a beamsplitter cube (Thorlabs BS014) which enables the pump beam's power to be measured by a power meter (Thorlabs PM100D). For each power increment, signal and background photon counts were measured for 500 ms for both the metalens and objective paths. The background countrates, plotted in Fig. 15**b-c**, are subtracted from the signal countrates to yield the points plotted in Fig. 4**e** in the main text.

Autocorrelation

When we centered the FSM on the NV center to record photons for cross-correlation, we were collecting both the photons emitted from the NV center as well as photons from the background. To account for this background and correct for it, we need to examine the $g^{(2)}(\tau)$ function and its boundary conditions. Given an arbitrary correlation function mixed with Poissanian background, the measured correlated function, $g^{(2)}_{\text{measured}}(\tau)$, is related to the ideal, background-free correlated function, $g^{(2)}_{\text{ideal}}(\tau)$, in the following way:

$$g^{(2)}_{\text{measured}}(\tau) = 1 - \rho^2 + \rho^2 g^{(2)}_{\text{ideal}}(\tau) \tag{S5}$$

this adjusted the boundary conditions of $g^{(2)}_{\text{measured}}(\tau)$ to the following:

$$g^{(2)}_{\text{measured}} = \begin{cases} 1 - \rho^2 & \tau = 0 \\ 1 & \tau = \infty \end{cases} \tag{S6}$$

where ρ is defined as:

$$\rho = \frac{S}{S+B} = 1 - \frac{B}{S+B} \tag{S7}$$

where S is the signal and B is the background. Both Eqn. S5 and Eqn. S7 make the assumption that the background in the measurement is Poissanian. To justify this assumption, we

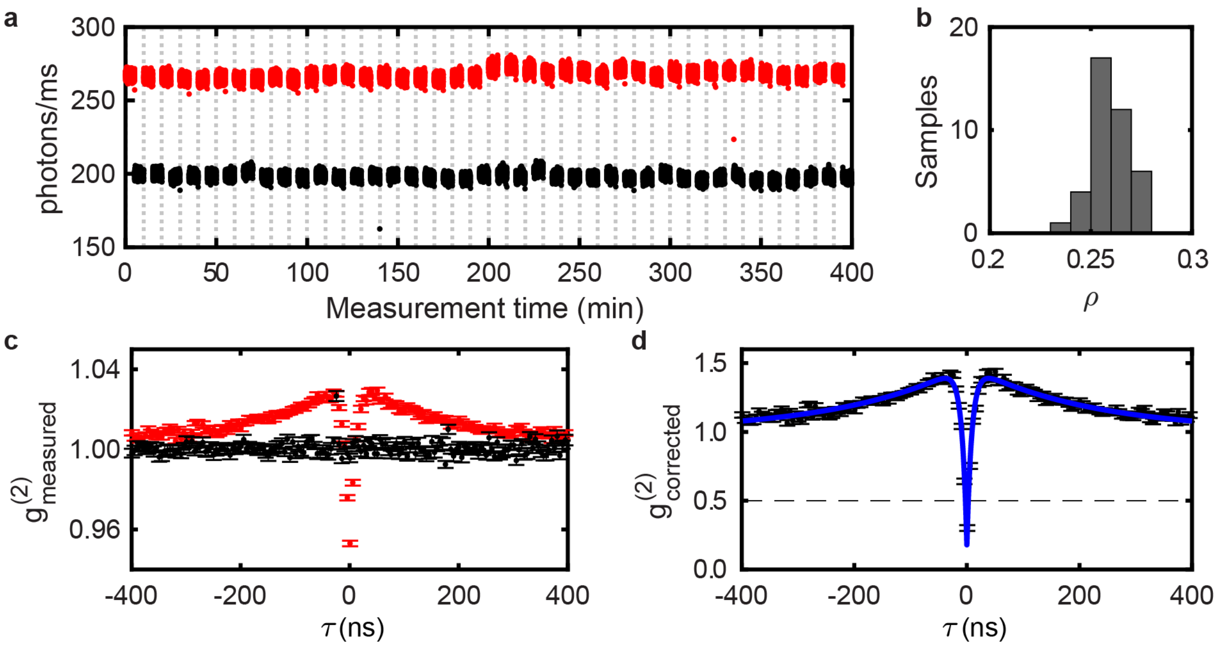

Supplementary Figure 16. **Background subtraction and fitting for autocorrelation measurements.** **a**, Total countrates (photons/ms) as a function of overall measurement time, where each pair of experiments, separated by dashed lines, comprised of an on-NV measurement (red) and off-NV measurement (black). The countrates plotted here represent the sum of photons from both collection paths. **b**, Histogram of signal-to-background ratio, ρ, calculated from each pair of experiments described in **a**, using Eqn. S7. **c**, Cross-correlation of photons collected during on-NV measurements (red) and off-NV measurements (black). **d**, Background-corrected cross-correlation with antibunching below the single-emitter threshold at $\tau = 0$ and bunching characteristic of an NV center, fitted to a 3-level system correlation function. Error bars in **c,d** represent the Poissan uncertainty in each bin of the correlation function.

moved the FSM to a spot off the NV center that is still within the metalens' field of view and measured photons from both metalens and objective paths for the same duration as we did for when the FSM is centered on the NV center (5 minutes). The off-NV (background) measurement was performed immediately following the on-NV (signal) measurement and the pair of measurements was repeated for 40 times. The recorded countrates are shown in Fig. 16**a** illustrating the consistency and stability of countrates over more than six hours of measurements.

We calculate ρ for each pair of experiments by using Eqn. S7 where B is measured as

countrates from off-NV measurements and $S + B$ is measured as countrates from on-NV measurements. The distribution of these ρ values is plotted in Fig. 16**b**.

Next, we calculate the cross-correlation of the recorded photons in the signal as well as the background measurements, shown in Fig. 16**c**. We use a variant of the algorithm developed by Laurence *et al.*[25], to calculate the cross correlation function from the raw photon arrival times. These measurements clearly demonstrate that the background is Poissonian, whereas the background-incorporated signal measurements showed cross-correlation characteristic of a single- or few-photon emitter.

To perform the background correction for $g^{(2)}_{\text{measured}}(\tau)$, we rearrange Eqn. (S5) to:

$$g^{(2)}_{\text{background-corrected}}(\tau) = \frac{g^{(2)}_{\text{measured}}(\tau) - (1-\rho^2)}{\rho^2} \tag{S8}$$

which yields the points plotted in Fig. 16**d** and in the main text.

We fit the background-corrected autocorrelation function using the well-known approximation of the NV center as a 3-level system[26]:

$$g^{(2)}_{\text{background-corrected}}(\tau) = 1 - Ae^{-\frac{|t-t_0|}{\tau_1}} + Be^{-\frac{|t-t_0|}{\tau_2}} \tag{S9}$$

where ideally $A = B + 1$ but we allow for the possibility of $A < B + 1$ to account for imperfect background measurements and finite detector bandwidth. The results of this fit is plotted in Fig. 16**d**, with $g^{(2)}(0) = 0.17 \pm 0.03$, which surpasses the single-emitter criterion of $g^{(2)}(0) < 0.5$ by 10.5 standard deviations. Furthermore, the fit demonstrates the characteristic short-delay bunching of an NV-center due to shelving in the spin-singlet manifold.

SUPPLEMENTARY REFERENCES

[1] Paniagua-Dominguez, R. *et al.* A metalens with a near-unity numerical aperture. *Nano letters* **18**, 2124–2132 (2018).

[2] Castelletto, S. *et al.* Diamond-based structures to collect and guide light. *New J. Phys.* **13**, 025020 (2011).

[3] Jamali, M. *et al.* Microscopic diamond solid-immersion-lenses fabricated around single defect centers by focused ion beam milling. *Rev. Sci. Instrum.* **85**, 123703 (2014).

[4]Siyushev, P. *et al.* Monolithic diamond optics for single photon detection. *Appl. Phys. Lett.* **97**, 241902 (2010).

[5]Zhang, Y. *et al.* Demonstration of diamond microlens structures by a three-dimensional (3d) dual-mask method. *Opt. Express* **25**, 15572–15580 (2017).

[6]Lalanne, P. & Chavel, P. Metalenses at visible wavelengths: past, present, perspectives. *Laser & Photonics Reviews* **11** (2017).

[7]Groever, B., Chen, W. T. & Capasso, F. Meta-lens doublet in the visible region. *Nano Lett.* **17**, 4902–4907 (2017).

[8]Johnson, S. G. & Joannopoulos, J. D. Block-iterative frequency-domain methods for maxwells equations in a planewave basis. *Opt. Express* **8**, 173–190 (2001).

[9]Liu, V. & Fan, S. S^4: A free electromagnetic solver for layered periodic structures. *Comput. Phys. Commun.* **183**, 2233–2244 (2012).

[10]Rumpf, R. C. Improved formulation of scattering matrices for semi-analytical methods that is consistent with convention. *Prog. Electromagn. Res. B* **35**, 241–261 (2011).

[11]Electron scattering and process effects quantified. `http://www.genisys-gmbh.com/web/assets/downloadInfos/TRACER_2017_WEB.pdf`. Accessed: 2017-10-30.

[12]Bickford, J. R., Lopez, G., Belic, N. & Hofmann, U. Hydrogen silsesquioxane on SOI proximity and microloading effects correction from a single 1D characterization sample. *J. Vac. Sci. Technol. B Nanotechnol. Microelectron.* **32**, 06F511 (2014).

[13]Electron- and laser-beam lithography software. `http://www.genisys-gmbh.com/web/assets/downloadInfos/Beamer_2017_web.pdf`. Accessed: 2017-10-30.

[14]Visser, T. & Oud, J. Volume measurements in three-dimensional microscopy. *Scanning* **16**, 198–200 (1994).

[15]Mildren, R. P. Intrinsic optical properties of diamond. In Mildren, R. P. & Rabeau, J. R. (eds.) *Optical Engineering of Diamond*, chap. 1, 1–34 (Wiley Online Library, 2013).

[16]Novotny, L. & Hecht, B. *Principles of nano-optics* (Cambridge university press, 2006).

[17]Corle, T. R. & Kino, G. S. *Confocal scanning optical microscopy and related imaging systems*, chap. 3 (Academic Press, 1996).

[18]Sheppard, C. J. R. & Török, P. Effects of specimen refractive index on confocal imaging. *J. Microsc.* **185**, 366–374 (1997).

[19]Chen, W. T. *et al.* Immersion meta-lenses at visible wavelengths for nanoscale imaging. *Nano Letters* **17**, 3188–3194 (2017).

[20] West, P. R. *et al.* All-dielectric subwavelength metasurface focusing lens. *Optics express* **22**, 26212–26221 (2014).

[21] Lu, B.-R. *et al.* Dielectric fresnel zone plates on optical fibers for micro-focusing applications. *Microelectronic Engineering* **88**, 2650–2652 (2011).

[22] Plakhotnik, T., Moerner, W., Palm, V. & Wild, U. P. Single molecule spectroscopy: maximum emission rate and saturation intensity. *Opt. Comm.* **114**, 83–88 (1995).

[23] Zheng, J., Liapis, A. C., Chen, E. H., Black, C. T. & Englund, D. Chirped circular dielectric gratings for near-unity collection efficiency from quantum emitters in bulk diamond. *Opt. Express* **25**, 32420–32435 (2017). URL `http://www.opticsexpress.org/abstract.cfm?URI=oe-25-26-32420`.

[24] Han, K. *et al.* Dark state photophysics of nitrogen-vacancy centres in diamond. *New Journal of Physics* **14** (2012).

[25] Laurence, T. A., Fore, S. & Huser, T. Fast, flexible algorithm for calculating photon correlations. *Opt. Lett.* **31**, 829–831 (2006). URL `http://ol.osa.org/abstract.cfm?URI=ol-31-6-829`.

[26] Brouri, R., Beveratos, A., Poizat, J.-P. & Grangier, P. Photon antibunching in the fluorescence of individual color centers in diamond. *Opt. Lett.* **25**, 1294–1296 (2000).
